# Interaction data from the Copenhagen Networks Study

**DOI:** 10.1038/s41597-019-0325-x

**Published:** 2019-12-11

**Authors:** Piotr Sapiezynski, Arkadiusz Stopczynski, David Dreyer Lassen, Sune Lehmann

**Affiliations:** 1 DTU Compute, Technical University of Denmark, DK-2800 Kgs. Lyngby, Denmark; 2 Center for Social Data Science, DK-1353 Copenhagen, Denmark

**Keywords:** Complex networks, Scientific data, Computational science

## Abstract

We describe the multi-layer temporal network which connects a population of more than 700 university students over a period of four weeks. The dataset was collected via smartphones as part of the *Copenhagen Networks Study*. We include the network of physical proximity among the participants (estimated via Bluetooth signal strength), the network of phone calls (start time, duration, no content), the network of text messages (time of message, no content), and information about Facebook friendships. Thus, we provide multiple types of communication networks expressed in a single, large population with high temporal resolution, and over a period of multiple weeks, a fact which makes the dataset shared here unique. We expect that reuse of this dataset will allow researchers to make progress on the analysis and modeling of human social networks.

## Abstract (summary)

**Table:** 

Measurement(s)	Call • Text Message • facebook friendship • Proximity
Technology Type(s)	mobile phone • social network analysis • bluetooth
Factor Type(s)	timestamp • sex
Sample Characteristic - Organism	Homo sapiens
Sample Characteristic - Environment	university campus
Sample Characteristic - Location	Kingdom of Denmark

Machine-accessible metadata file describing the reported data: 10.6084/m9.figshare.11283407

## Background & Summary

The purpose of collecting the Copenhagen Networks Study (CNS) dataset was to accelerate our understanding of social systems. In particular, we were interested in the following major topics: Measuring networks across modes of communication; Modeling temporal social networks; Modeling spreading processes on social networks; Analyzing and modeling human mobility; Understanding the interplay between mobility and social behavior; Privacy.

Because of our focus on understanding social networks, we enrolled a group of participants (more than 700 freshmen at the Technical University of Denmark) likely to constitute a highly interconnected network. Due to the scale of the study, the amount of raw data collected was substantial: each participant uploaded between 50–100 megabytes of data per day, resulting in new data per day in the range of 50 to 100 gigabytes.

Here, we cannot share the entire raw dataset, below we motivate our choice of which selection of data to publish. The privacy of the study participants is central in the Copenhagen Networks Study, as documented through the study design^1^, as well as our work on privacy discussed in the next section. In a complex dataset, such as ours, it is virtually impossible to provide guarantees regarding re-identification of users while preserving its value for the stated research purposes^2^. In preparing the data for publication, it was therefore necessary to restrict the data released as described below to make de-anonymization as difficult as possible, but without compromising the dataset’ usefulness for research. In addition to obstructing de-anonymization, each step listed below serves the second purpose of limiting the potential harm to data subjects in the unlikely case of a successful re-identification attack.

- *Limiting the types of data available*. As part of Copenhagen Networks Study we collected information beyond the dataset we make available here, such as location traces and WiFi logs. Such data-types carry high risk of re-identification through publicly available information. In Denmark, for example, the physical address of every citizen (and phone number) is by default public and published in an open index. Further, geo-located tweets or Instagram posts, social physical activity app data, etc can also be used to re-identify geospatial data. To avoid this attack, we limit our release to information that cannot be easily cross-correlated with public datasets.
- *Limiting the timespan of released information*. The data released only have relative time-stamps. This makes it difficult to cross-correlate the released data with external information. Releasing a full year of data would make it trivial to identify holidays and reconstruct the absolute timestamps. Thus, we do not publish the absolute starting time of the data, but note that the dataset starts on a Sunday during school term.
- *Delaying the data release*. In the European Union, phone metadata retention is limited to two years. By waiting beyond the retention period we limit the probability that a person with access to the network operator data (CDR, call detail records) can use such data to re-identify individuals in our dataset. A similar threat is why we do not release Facebook activity data, which could also be used to re-identify individuals via data from inside Facebook (which retains data indefinitely). Furthermore, at the same time, in the case of re-identification, older data is less harmful as it is less likely to be a precise reflection of data subjects’ current social networks and behaviors, and even more so since students have moved on from university. In terms of research, this information from a few years ago is just as useful as at the time of collection.

Because the data was recent at the time of publishing most of our research, we took a cautious approach and did not release it then. We are only able to release it now, after a careful balancing of threats and research usefulness.

These considerations on privacy versus types of data released also impact our view on reproducibility given this dataset. The aim of this data release is first and foremost to enable use of rich multi-layer network data for new work, while still respecting participant privacy. That being said, however, much of the work published so far from this dataset was similarly based on four-week subsets (which might or might not overlap with the provided time period). Thus, the timespan of the data is not a limitation to replicability of already published work.

In addition to the network data we release in this paper, the overall CNS study collected detailed high-resolution GPS location (sampled every 5 minutes), information on nearby WiFi routers and cellular towers, screen on/off status, battery charge level, as well as demographic and questionnaire information on all participants^1^. The questions in the 2013 deployment included The Big Five Inventory^3^, Rosenberg Self Esteem Scale^4^, Narcissism NAR-Q^5^, Satisfaction With Life Scale^6^, Rotters Locus of Control Scale^7^, UCLA Loneliness scale^8^, Self-efficacy^9^, Cohen’s perceived stress scale^10^, Major Depression Inventory^11^, The Copenhagen Social Relation Questionnaire^12^, and Panas^13^, as well as health- and behavior-related questions.

A dataset describing human behavior with the richness captured in the CNS study, inevitably raises questions of privacy and personal data. In the CNS data collection, privacy was therefore not only important for the sake of participants, but also an active area of research. In collecting the data, we also had to answer the question, ‘how can we work on these data while respecting the privacy of the study participants?’. Therefore, we now briefly discuss overall privacy concerns and challenges. The research project and data collection was registered with and approved by the Danish Data Supervision Authority before data collection commenced. All data was collected with informed consent and with every participant able to withdraw from the study and have their data deleted. This protocol, implemented in 2012 and 2013, was in effect similar to the rules being introduced with the EU General Data Protection Regulation (GDPR) which came into effect in May 2018. In the present release, to comply with GDPR, the data has been stripped of personally identifying information and the data has been reduced in such a way that there is no reasonable likelihood of re-identification occurring.

Details on the actual implementation and broader philosophy of ensuring privacy in sensor-driven human data collection can be found in^1^ and^14^. Here, we will remark on two integrated components of the privacy strategy. First, it is well known that formal informed consent can be insufficient to meet actual privacy demands as construed by participants^15^. To address this, we, in addition to the written informal consent paragraph, conducted numerous presentations of the project to students before they signed up, published blog posts, and answered questions using Facebook. Second, we designed a ‘quantified self’ module allowing participants to access and visualize their own – and only their own – data traces^16^. As the students interacted with these tools, the they were able to develop a better understanding of the nature and the depth of the collected data, thus making their consent more informed, or – as was the case for a single student – choose to withdraw from participation.

Our work on collecting data from smartphones does not stand alone. In the section below, we provide a brief overview of related work. Mobile phones have been a source of data on human activity and interaction since the early 2000s. They have been used to collect data broadly (coarse grained, sampled data describing millions of individuals) or deeply (fine grained data on fewer individuals). On the large scale, teams have studied the connections between individuals on a societal level in Belgium and Great Britain^17,18^, as well as the mobility of millions of individuals^19,20^. At the other end of the spectrum, teams from MIT’s MediaLab have pioneered highly detailed studies of smaller populations. The landmark study is the *Reality Mining dataset*^21^, but more recently many updated studies have been published, in part run by teams at MIT^22–25^ as well as the Nokia Research Center in Lausanne^26^ and Aalto University^27^. Other similar technologies for measuring social networks have also been developed, for example based on RFID tags, and provide an important alternative to cellphones as social sensors^28–31^. In terms of size, CNS increased the number of participants by almost a full order of magnitude compared to state-of-the-art high-resolution studies^21,25,26^.

Finally, we address the dataset’s potential for reuse. The dataset collected as part of CNS has already been used for research in a number of areas. There is a number of publications covering technical aspects of data collection and analysis^16,32–35^. Another set of papers focused on modeling and analyzing network structure^36–41^, epidemiology^42–44^, as well as work on human mobility^45–50^, and privacy^14,46^. Additionally, there is a body of work that goes beyond the stated goals of the CNS project. For example, researchers studied behavioral differences between the two sexes^51,52^, along with studies on academic performance^52–54^, activity patterns^55^, sleep patterns^56–58^, and much more.

The broad and varied research that has already been published based on this dataset underscores its richness. Given that we are only able to release network data, we expect reuse of this dataset to focus on the modeling and analysis of multi-layer temporal networks and we hope that the data released here will allow researchers to make progress on understanding human social networks.

## Methods

**Fig. 1:**
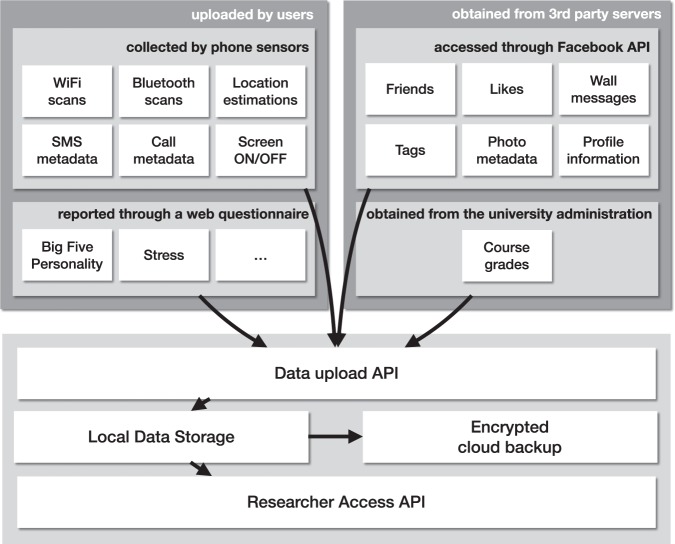
A schematic view of the data collection for CNS. See main text for a detailed description and Stopczynski *et al.*^1^ for a full overview.

The Copenhagen Networks Study accumulated data from a number of channels: smartphones, online questionnaires, and third parties. The data collection system was designed to ensure privacy of the participants and maintain access control to the data, and is described in detail in Stopczynski *et al.*^1^. Figure 1 presents a simplified overview of the system. Data from all sources were collected first on a central on-premises server. The data were then stripped of personally identifiable information and replicated both locally and as an encrypted cloud backup. Pseudononymized data were then made available to approved researchers via access-controlled API. In this work we make a subset of this dataset available to the public for the first time^59^.

In the sections below we explain the details of the collection methods for each channel.

### Smartphone data collection

Each participant in the study was equipped with an Android-based Google Nexus 4 smartphone and required to install the data collection software from Google Play Store. Participants agreed to use this study issued devise as their primary phone. The data collection software was based on the Funf platform (http://www.funf.org/); its source code along with all the modifications we introduced, is open and available on Github (https://github.com/OpenSensing/funf-v3). The software triggered data collection from multiple channels: cell phone location (from AGPS), nearby cell towers, WiFi routers, and Bluetooth devices, as well as collected this information whenever another application on the phone requested it from the system. Additionally, each day we collected the meta-data logs of calls and short messages. Our previous work provides a full description of the collected data^1^, here we focus on the three channels which we now make available to the public: Bluetooth data as well as call and SMS metadata. In terms of node-metadata, we include the gender of each participant.

### Bluetooth data

Bluetooth is a wireless communication standard designed to provide connectivity over distances of up to 10 m (30 ft). Each device in the experiment was configured to be *discoverable* at all times, and to *discover* nearby Bluetooth devices every five minutes. During the discovery process (or *scanning*), a device sends probe requests and receives responses from all nearby Bluetooth discoverable devices. Each response contains a unique identifier of the discoverable device, which also uniquely corresponds to the study participant carrying the device, enabling us to track proximity events between the study participants. Additionally, the device measures and reports the Received Signal Strength (RSSI), which can be (roughly) mapped to physical distance: a high RSSI means that the two devices are physically close, a low measure indicates that they are further apart or that there are obstacles inbetween. In previous work we investigated the interplay between the distance and RSSI in detail^32^.

To prepare the data for release we followed the same pre-processing steps as in other work we published based on this dataset (e.g.^36,42^):

1. We removed identifiers belonging to discovered devices that were not in the experiment, and mapped the participating devices’ identifiers to their users.
2. We quantized the time of each scan into bins of five minutes.
3. Within each timebin we found all instances of users *A* and *B* discovering each other, reported the one with the highest RSSI, and discard others.
4. The information of directionality (whether user_*A*_ discovered user_*B*_ or vice versa) is discarded.
5. In bins where user_*A*_ was actively scanning, but found no other Bluetooth devices in proximity, we reported the alter ID as −1 and the received signal strength as 0.
6. In bins where user_*A*_ discovered other Bluetooth devices but not other study participants, we reported the alter ID as −2 and the highest received signal strength measured. We do not report the type of the discovered device.

The data is presented as a temporal, weighted edge list, and each edge is described using (1) the timestamp of the beginning of the timebin in seconds (because of the quantization of time into five minute bins the timestamp is reported in the multiples of 300 seconds), (2, 3) the IDs of users who discovered each other, (4) the measured received signal strength. Note, that in some of the published work (e.g.^36^,) we performed the additional step of triadic closure, i.e. if user_*A*_ discovered user_*B*_ and user_*B*_ discovered user_*C*_, we assumed proximity between user_*A*_ and user_*C*_ regardless of whether they discovered one another. Since there is no meaningful RSSI to assume in such cases, we do not perform this step here and instead we leave it to the researchers using this data to decide which approach is appropriate for their specific analysis.

### Calls and short messages

Call and message logs were obtained from the smartphones every day. For privacy reasons, we did not capture or store the content of the interactions, only the metadata. Since the participants were required to use the provided smartphones as their primary phones and to reveal their phone number, we matched the entries in the call logs to the participants’ identities. Each record in the call logs is in the form of timestamp, user_*A*_, user_*B*_, and call duration (in seconds). Each record in the SMS logs is in the form of timestamp, user_*A*_, and user_*B*_. In both cases the data is organized such that user_*A*_ initiates the interaction and user_*B*_ is the recipient.

### Facebook data

Most of the participants of the study voluntarily opted in to authorize data collection from Facebook. We used the official Facebook API and the access tokens provided by participants to collect their Facebook data every day. The data we collected include all the participants’ activity, characteristics, and the contents of their News Feed. Here, we release a static snapshot of the friendship network among the participants at the end of the observation period (links to non-participants are removed). This friendship network is presented in the form of a static edge list.

### Data quality

**Fig. 2:**
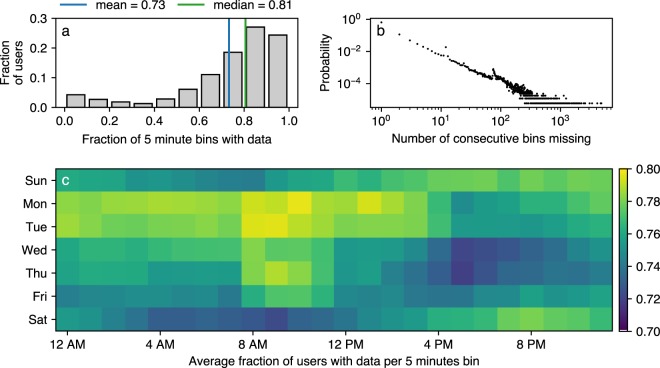
Statistics on data quality. (**a**) Missing data per user. The data availability – measured as the fraction of 5 minute timebins in which data is available – varies across users. Half of the users have at least 81% of data available. (**b**) Distribution of time-bins with missing data. Most commonly only a few bins are missing. The visible peak at 12 bins, corresponds to 1 hour of data—the interval at which the phones moved collected data into encrypted files—and could be caused by file corruption on the device. (**c**) Data availability across the week. The data availability is the highest during working hours, when the majority of interactions occur.

In this section we report on the quality (availability) of the Bluetooth data. Phones in the experiment were set to scan for Bluetooth every five minutes. We therefore divide the four weeks observation period into 8064 five minute periods (4 (weeks) × 7 (days) × 24 (hours) × 12 (five minute periods) = 8064). The data quality (availability) of each user is the fraction of these periods in which they have scan data (there were actively scanning and/or scanned by another user). Figure 2 summarizes the Bluetooth data availability concerns. Panel a displays the distribution of data quality as defined above: the median availability is 0.81, meaning that half the users have data in 81% of timebins or more. In Panel b, we show that in most cases only few consecutive bins are missing, with a small peak at one hour. Panel c emphasizes the fact that missing data is correlated in time. The best quality is observed during working hours and the worst on the night between Friday and Saturday. We expect higher date coverage when more users are interacting – even if one user’s phone fails to report scan results, that user is likely to be scanned by others around them. The effect of the Friday night missing data is a combination of fewer study participants nearby each other and the study participants neglecting to charge their phones during a night out.

## Data Records

All data is available in Figshare^59^. A description of the Bluetooth interaction data is available in Table 1, call info is listed in Table 2, text message data descriptions are in Table 3, the Facebook data description is in Table 4, and description of the gender information is stored in Table 5.

**Table 1:** Bluetooth interactions. These are listed in bt.csv and are formatted as described above.

column name	column description
timestamp	Timestamp in seconds from the beginning of the observation period (as reported by the device. Note, that because of differences in the internal clock of different devices, some of the measurements will not be perfectly aligned.)
user_a	ID of one user (ego).
user_b	ID of the other user (alter). 0–850 for participants of the study, −1 for empty scans, −2 for any non-participating device.
rssi	Received Signal Strength Indication, measured in dBm, a rough proxy for distance between devices (the higher the absolute value, the higher the distance)
**Summary**: 5,474,289 records, 706 total users

**Table 2:** Phone calls. These are stored in calls.csv and are formatted as described above.

column name	column description
timestamp	Timestamp in seconds from the beginning of the observation period
user_a	ID of the user initiating the call
user_b	ID of the other user receiving the call.
duration	Duration of the interaction in seconds, or −1 for missed calls
**Summary**: 3,600 records, 540 total users

**Table 3:** SMS data. Short messages are listed in sms.csv are formatted as described above.

column name	column description
timestamp	Timestamp in seconds from the beginning of the observation period
user_a	ID of the user sending the message.
user_b	ID of the user receiving the message (non-participants were removed).
**Summary**: 24,333 records, 577 total users

**Table 4:** Facebook friendships. The static snapshot of Facebook friendships is stored fb_friends.csv, and formatted according to the description in this table.

column name	column description
user_a	ID of user A
user_b	ID of user B
**Summary**: 6,429 edges, 800 total users

**Table 5:** Binary descriptors of participants’ gender. These are recorded in genders.csv, and formatted as explained above.

column name	column description
user	ID of user
gender	gender of user; 0 for male, 1 for female
**Summary**: 788 total users	

## Technical Validation

In this section we describe and visualize the properties of the networks, providing readers with an overview which we hope will facilitate working with the data.

### Person to person proximity (Bluetooth data)

#### Temporal dynamics

**Fig. 3:**
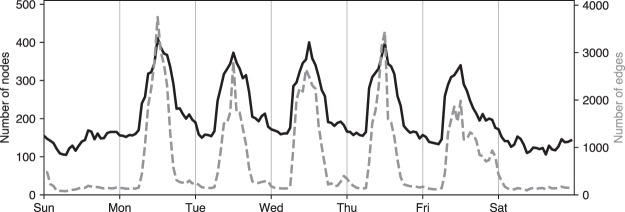
Bluetooth activity reveals a clear circadian and weekly rhythm. Black solid line shows the number of active nodes, while the dashed gray bars show the number of active edges.

The properties of the presented Bluetooth data reflect the highly dynamic and circadian nature of interactions between the participants of the study. Figure 3 summarizes 168 hours (one week) of data by reporting the number of active participants (i.e. participants in the vicinity of other participants) as well as the number of active edges in the network. Note that a small part of the network is active over night and during weekends: some students share accommodations or have dorm rooms adjacent to one another. During daytime on weekdays, the network grows and becomes much more connected. Notice as well, that the overall properties of the network during Friday are different than other days of the week, with interactions continuing late into night hours.

#### Temporal aggregates

**Fig. 4:**
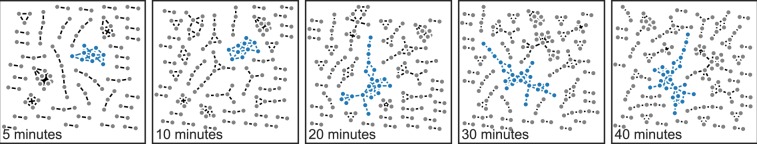
Temporal aggregation of the Bluetooth network. The biggest connected component (shown in blue) grows steadily as we increase the duration of the aggregation window from five minutes—the underlying sampling frequency in the data—to 40 minutes.

**Fig. 5:**
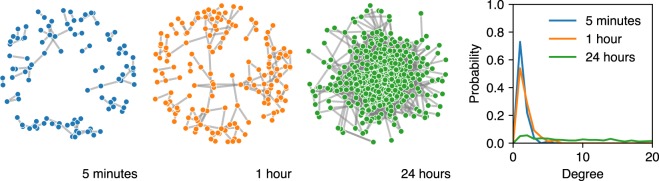
Temporal aggregation of the Bluetooth network. By using daily aggregates important structures are obscured^36^.

Given the volume and high temporal resolution of the Bluetooth data, one might introduce temporal aggregation to simplify the analysis of the data. It is, however, important to note that the structure of the network changes drastically as the aggregation window grows. Figures 4 and 5 illustrate the effects of aggregation. At five minutes—the underlying temporal resolution of the data—participants form multiple small, disjoint groups. Figure 4 highlights how the biggest connected component in the graph grows with aggregation up to 40 minutes. Figure 5 shows the effect of aggregating the data for up to 24 hours: the structure of the network is not clear from the network layout, and the average degree grows from ∼2 to ∼30. For more details on how aggregation changes the structure of the network and the subsequent implications for epidemic modelling, refer to Stopczynski *et al*.^43^. For a more detailed description of the network structures at the highest resolution, and the implications for modeling of social interactions, refer to Sekara *et al*.^36^.

**Fig. 6:**
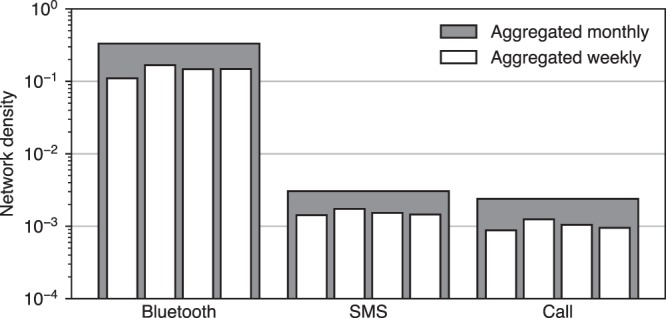
Relative network density in weekly and monthly aggregates. Density is the fraction of dyads that are active vs the number of possible dyads. In the nested bar-charts, the dark gray bars show data aggregated by month, while light bars show data aggregated weekly.

Figure 6 shows that the aggregates of the Bluetooth networks are very dense: in the weekly aggregates between 10% and 20% of possible links are active at least once, and during the entire observation 30% of all possible links were active.

### Telecommunication network

**Fig. 7:**
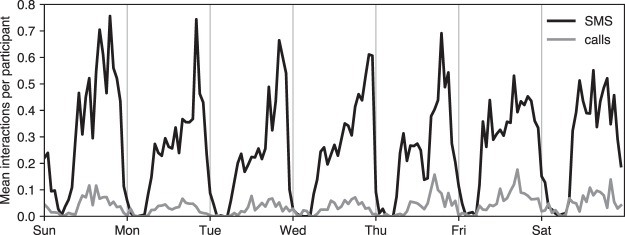
Telecommunications activity. Contrary to interactions in the physical space, the participants call/text each other most during evenings and weekends. The black solid line shows the mean number of text messages per participant as a function of time, whereas the gray line shows the corresponding mean number of phone calls.

Figure 7 shows that the participants of the study prefer text messages to making phone calls, with an order of magnitude higher number of SMSes than calls. The telecommunication networks appear to be complementary to the person-to-person network: participants resort to text messages and phone calls in more the evenings and weekends, when there are fewer proximity events. As shown in Fig. 6, the difference in the density of aggregate networks is not pronounced as strongly as in the sheer volume of communications. There is a positive correlation between the number of messages and phone calls dyads exchange.

### Network comparisons

**Fig. 8:**
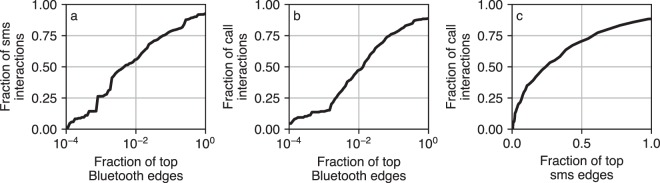
Overlap between most active dyads in different networks. Dyads who interact a lot in physical space also communicate most through calls and sms. 1% of most active Bluetooth dyads correspond to (**a**) 56% short messages exchanged and (**b**) 47% of phone calls. (**c**) 1% of most active sms dyads correspond to 6% of phone calls.

The overlap between networks is an interesting avenue of investigation, which we explored to some extent in our previous work^32,38,40^. Figure 8 shows the overlap between most active dyads in different networks. We see that over 80% of short messages are exchanged among 15% of dyads that have the most physical proximity (panel A), and that 89% of dyads call each other are also in physical proximity at least once during the observation (panel B).

### Data loading

The data is released as CSV files. We show how data can be loaded using the Pandas^60^ package in Python. All data files are directly loadable with a basic call of pandas.read_csv().

### Bluetooth network visualization

The iPython notebook released with the data shows a visualization (using NetworkX^61^ and Matplotlib^62^) of the temporal Bluetooth network by considering the network structure at a single 5-minute bin, similar to the visualization in Fig. 5. At this high temporal resolution, the network consists of many small connected components which can be directly used for network analysis^36^.

We note that while the network is prepossessed to be symmetric wrt. RSSI values (we store the higher value of RSSI between two users), the components in the network are not necessarily fully connected: if user_*A*_ saw user_*B*_ and user_*C*_, but user_*B*_ did not see user_*C*_ (nor vice versa) we do not create a link between user_*B*_ and user_*C*_. Due to high temporal and spatial resolution of the provided data, users of the of data may consider treating the components as fully connected at given time slice, expecting that spurious connections disappear in analysis over longer periods, see, for example, Sekara *et al*.^36^.

### SMS communication visualization

The notebook also shows the principle of joining different types of data by visualizing the number of text messages sent between users of different genders in the study. Using a consistent user id in all the data types, allows for straightforward merging of different subsets, allowing us to, for example, consider dynamics of communication separately for different genders.

## Data Availability

Alongside the data, we provide an iPython notebook showing basic data loading and use (see Copenhagen_Networks_Study_Notebook.ipynb in the Figshare data repository^59^). The notebook is intended to showcase the basic approaches to working with the released data.
